# Conservative Surgical Management of Adenomyosis: Implications for Infertility and Pregnancy Outcomes—A Perspective Review

**DOI:** 10.3390/jcm14196956

**Published:** 2025-10-01

**Authors:** Alexandra Ioannidou, Konstantinos Louis, Dimos Sioutis, Periklis Panagopoulos, Charalampos Theofanakis, Nikolaos Machairiotis

**Affiliations:** Third Department of Obstetrics and Gynecology, National and Kapodistrian University of Athens Medical School, Attikon Hospital, 1 Rimini, 124 62 Athens, Greece; kostaslouisss@gmail.com (K.L.); dsioutis@gmail.com (D.S.); paninosrafaela@yahoo.gr (P.P.); ch.theofanakis@gmail.com (C.T.); nikolaosmachairiotis@gmail.com (N.M.)

**Keywords:** adenomyosis, fertility, infertility, pregnancy outcomes, adenomyomectomy, conservative surgery, uterine-sparing surgery

## Abstract

**Background/Objectives:** Adenomyosis is increasingly being identified in women of childbearing age as a cause of infertility and adverse pregnancy outcomes. As hysterectomies are not suitable for fertile women, conservative surgical management has become a promising solution. We aimed to synthesize current evidence on conservative uterus-sparing surgical techniques for adenomyosis, focusing on implications for infertility treatment and pregnancy outcomes. **Methods:** A search of PubMed, Google Scholar, and Europe PMC from 2022 to July 2025 was conducted using combinations of the words “adenomyosis,” “fertility,” “infertility,” “pregnancy outcomes,” “adenomyomectomy,” and “uterine-sparing surgery.” Sixteen high-relevance studies were chosen that included reproductive-aged women who had conservative surgery for adenomyosis. **Results:** Excisional techniques such as adenomyomectomy yield pregnancy rates of >50% and live birth rates of up to 70% in focal disease, with less success in diffuse disease. Non-excisional approaches—high-intensity focused ultrasound (HIFU), radiofrequency ablation (RFA), and uterine artery embolization (UAE)—yield equivalent pregnancy (40–53%) and live birth (35–74%) rates in selected patients, with fewer surgical complications. Adjunctive hormonal therapy, particularly GnRH agonists, appears to improve outcomes. Risks include placenta accreta spectrum disorders and uterine rupture (≤6%), especially in diffuse adenomyosis. The type of lesion, location, and junctional zone thickness are strong predictors of fertility outcomes. **Conclusions:** Conservative surgery can augment fertility in appropriately chosen women with adenomyosis, with removal being the preferred treatment for focal disease and non-removal techniques offering encouraging alternatives in mild or intracorporeal disease. The addition of adjunct hormonal therapy and standardized patient selection criteria will optimize results. The lack of European professional society guidelines underscores the need for harmonized protocols in order to standardize the diagnosis, surgery, and reporting of results.

## 1. Introduction

Adenomyosis is a benign uterine condition. It is characterized by the presence of endometrial glands and stroma within the uterine muscle layer. The symptoms are painful periods, heavy bleeding, and, interestingly, infertility. Historically, this condition would be diagnosed in women aged 40 to 50 years. Despite advances in imaging, like MRI and high-resolution transvaginal ultrasound, diagnosing adenomyosis remains challenging due to the absence of standardized imaging criteria.

Some of the biologic mechanisms whereby adenomyosis may lead to infertility include interference with uterine movement, chronic inflammation with alteration in cytokine and estrogen signaling, oxidative stress, and compromised endometrial receptivity. These can all disrupt implantation and embryo transport [1]. It has been demonstrated that adenomyosis can reduce clinical pregnancy rates to 40–46%. It also increases the risk of miscarriage by a significant degree and reduces live birth rates in assisted reproductive technology (ART) settings [2].

For women who would like to preserve fertility, hysterectomy is not an appropriate option. Uterus-sparing surgeries have, therefore, been investigated. These include focal adenomyomectomy and others like wedge resection, overlapping flap techniques, H-incision, and double-flap reconstructions. These surgeries are aimed at improving reproductive outcomes without making the uterus nonfunctional [3].

In a cohort of over 1300 infertile women who had undergone fertility-sparing surgery, pregnancy rates were approximately 52.7% for focal adenomyosis and 34.1% for diffuse types, with a miscarriage rate of approximately 21% in each group. The risk of uterine rupture, especially after surgery for diffuse adenomyosis, was reported to be as high as 6.8%. In contrast, no ruptures were seen with focal adenomyosis [4].

In a cohort study from the JSOG (Japan Society of Obstetrics and Gynecology) registry, adenomyosis was found to be an independent risk factor for uterine rupture, placental complications, and fetal growth restriction [5].

Despite reporting favorable outcomes for fertility results, the literature is not devoid of limitations. These include small sample sizes, retrospective designs, variable surgical approaches, and heterogeneous reporting of the disease subtype and severity. There is also a significant lack of randomized controlled comparisons between surgery and medical or expectant management approaches.

## 2. Materials and Methods

This perspective review aimed to synthesize and critically assess the latest literature regarding conservative surgical management of adenomyosis and its effects on infertility and pregnancy outcomes. A systematic literature search was performed using PubMed, Google Scholar, and Europe PMC, selecting articles from 2022 through July 2025.

The search terms were pairs of “adenomyosis,” “fertility,” “infertility,” “pregnancy outcomes,” “adenomyomectomy,” “conservative surgery,” and “uterine-sparing surgery.”

We included original articles, systematic reviews, meta-analyses, and English-language cohort and case series. We preferred studies involving human subjects, particularly reproductive-aged females who were treated with conservative surgical interventions for adenomyosis. We excluded articles that took into account hysterectomy-only methods, animal model studies, or purely diagnostic research.

We collectively screened 108 articles and selected approximately 16 high-relevance studies, applying the inclusion and exclusion criteria referred to above. We focused on studies with clear diagnostic definitions and fertility-related outcomes, and we gave preference to well-documented clinical reports over studies with limited methodological detail in order to minimize the risk of bias.

## 3. Pathophysiology of Infertility in Adenomyosis

Current studies are increasingly exploring the molecular and immune foundations of infertility in adenomyosis patients. One of the significant findings suggests an abnormal immune cell population in the uterine microenvironment. Women with adenomyosis are likely to have a higher number of macrophages, a disturbed balance of the T-helper/Treg cell ratio, and upregulated production of inflammatory cytokines like TNF-α, IL-1β, and IL-6. Such an immune imbalance impairs endometrial receptivity by generating an unfavorable uterine microenvironment. This significantly decreases the likelihood of successful embryo implantation [6].

Concurrently, oxidative stress and inflammation in the microenvironment are also important. An excessive generation of reactive oxygen species (ROS) in the endometrial and follicular tissues due to immune activation and enzyme dysfunction damages oocytes and embryos. Certain major enzymes, such as xanthine oxidase, nitric oxide synthase, and superoxide dismutase, overfunction and fail to switch as they should with the advancement of the menstrual cycle. This increases ROS levels and harms early embryonic development. Oxidative stress, therefore, negatively impacts mitochondrial function, spindle formation, and DNA integrity, decreasing fertility despite ART [7].

These oxidative and immune problems, combined with structural changes in the junctional zone, contribute to defective myometrial contractions and increased uterine motion. Structural changes in the junctional zone obstruct uterotubal transport and generate abnormal pressure within the uterus that may interfere with the appropriate placement and implantation of embryos. Whenever this mechanical impairment accompanies molecular and cellular abnormalities, the result is a very high miscarriage rate [6].

Studies demonstrate that adenomyotic lesions exhibit enhanced estrogen and progesterone receptor expression, which links estradiol with enhanced cell proliferation and progesterone with prolactin secretion, thereby highlighting the central role of sex steroid signaling in adenomyosis pathophysiology [8]. Accordingly, the non-surgical management of adenomyosis includes hormonal treatments like GnRH agonists, progestogens, dienogest, and the levonorgestrel-releasing intrauterine system, which are found to decrease symptoms and, in some cases, improve fertility results, although evidence is limited and found predominantly in non-randomized or small studies [6].

## 4. Conservative Surgical Techniques

### 4.1. Excisional Surgery (Adenomyomectomy)

Supported by laparotomy, laparoscopy, or robotic procedures, adenomyomectomy focuses on resecting some lesions while preserving the uterus. Several reconstruction methods, such as triple-flap, overlapping flaps, or U- or H-suturing, have been utilized in an attempt to reduce defects and enhance the uterine wall [9]. Studies have shown that patients with focal adenomyosis experience greater benefits in comparison to patients with diffuse disease [4].

### 4.2. Non-Excisional Methods

These methods include uterine artery embolization (UAE), high-intensity focused ultrasound (HIFU), and microwave or radiofrequency ablation. They cause necrosis or ischemia of adenomyotic tissue but do not excise it directly. Evidence supporting fertility improvement is mixed [10].

Table 1 presents some practical aspects of each method regarding technology availability and the required operative skills.

## 5. Fertility Outcomes After Uterus-Sparing Surgery

Conservative surgeries preserving the uterus—primarily adenomyomectomy—have returned as fertility-sparing therapies for adenomyosis in patients who wish to become pregnant. In the past three years, new evidence—systematic reviews, prospective cohorts, and well-characterized single-center series—has illuminated reproductive outcomes, risks, and determinants of surgical success.

The most inclusive synthesis of the new data is the meta-analysis and systematic review of Jiang et al., which included 13 studies of 1319 women, 795 of whom were attempting conception. Excisional procedures—open, laparoscopic, or robot-assisted adenomyomectomy—had a total pregnancy rate of 40%, a cumulative miscarriage rate of 21%, and a live birth rate of 70%. Non-excisional treatments—HIFU, UAE, radiofrequency ablation (RFA), and microwave ablation—had the following outcomes: pregnancy 51%, miscarriage 22%, and live birth 71%. Much more importantly, excisional and non-excisional treatment outcomes did not have statistically significantly different fertility outcomes [10].

### 5.1. Excisional Techniques

High-quality prospective studies report a delivery rate of 46.9% (84/179) in patients who had received uterus-sparing surgery for adenomyosis. The conception rate was 58.1% (104/179), and the miscarriage and preterm birth rates were 8.9% (16/179) and 9.6% (10/104), respectively. Notably, none of these studies mentioned any uterine ruptures. However, because most existing studies are retrospective, with non-standardized mechanisms of determining conception and pregnancy outcomes and involving non-randomized patient selection, direct comparisons between different surgical methods remain imprecise [11].

In a retrospective study over several years, Wang et al. assessed pregnancy outcomes in patients with diffuse adenomyosis who were treated with uterus-sparing double-flap adenomyomectomy. Of 137 women who desired fertility, 62 pregnancies from 56 women were registered, 56.5% spontaneous and 43.5% after ART. From those 62 pregnancies, the live birth rate was 72.6%. Almost all births were delivered by cesarean section, except one, which was vaginal. According to ROC analysis, the most predictive factor was postoperative JZmax-A (junctional zone), which had 3-year cumulative pregnancy rates of 70.1% for JZmax-A ≤ 8.5 mm and 20.9% for >8.5 mm [12].

A retrospective case–control study compared 18 adenomyomectomy patients with 105 matched controls who had not received any treatment for adenomyosis. In the surgical group, second-trimester abortion was not observed (0% vs. 7.6%, *p* = 0.22). Postoperative patients also had significantly reduced rates of PPROM (preterm prelabor rupture of membrane), preeclampsia, and small-for-gestational-age neonates (each 0% vs. ~12–15%, *p* < 0.05). However, the surgical group also had a 50% incidence of placenta accreta spectrum, compared with none for controls, and one uterine rupture at 30 weeks (5.6%) [13].

A homogeneous single-surgeon series (*n* = 22) extended this. All women were delivered by cesarean section. A total of 31.8% had preterm delivery; four had placental pathology (two accreta, two previa), and one had uterine rupture at 27 weeks (4.5%). There were no hysterectomies, and neonatal outcomes overall were acceptable [14].

The first successful twin pregnancy following elastography-guided laparoscopic adenomyomectomy was reported in 2024. One woman with diffuse adenomyosis at the age of 35 conceived after IVF following the procedure. Real-time elastography permitted precise resection of the tissue while maintaining the integrity of the uterus. Pregnancy was achieved without severe complications, showing that this method can enhance fertility success rates and reduce complications of surgery, though this must be confirmed [15].

### 5.2. Non-Excisional Techniques

#### 5.2.1. HIFU

Xiong et al.’s retrospective analysis evaluated 27 women with primary infertility and adenomyosis who received HIFU. An interesting observation was a 37% pregnancy rate (PR), with 72% being spontaneous pregnancies, and a 90% rate of full-term delivery in patients with successful outcomes. The cesarean section rate was 50%. There were no severe complications at the time of delivery, and perinatal courses were otherwise normal. Multivariate analysis demonstrated that duration of infertility was the best predictor of successful pregnancy, emphasizing the importance of early treatment. Of special interest was the observation that the study demonstrated not only that HIFU improved uterine complaints such as dysmenorrhea and menorrhagia but also preserved reproductive potential with no compromise of safety [16].

As a supplement to these findings, the systematic review and meta-analysis of Chen et al. pooled data from 10 studies (*n* = 557) and reported an overall live birth rate (LBR) of 35.2% and an overall pregnancy rate (PR) of 53.4% after HIFU. The authors did have significant heterogeneity and methodological heterogeneity among the studies, including a lack of randomized controlled studies, possible conflicts of interest, and the prevalence of single-arm studies. There is evidence of a possibly exaggerated benefit in less stringent design trials, with a note of caution regarding not overestimating the present evidence [17].

Bahutair and Alhubaishi’s comprehensive review of HIFU for adenomyosis treatment consolidates evidence demonstrating its effectiveness in reducing both lesion and uterine volumes and alleviating associated symptoms such as dysmenorrhea and menorrhagia. Volume reduction rates ranged from 45% to 56% within 3–6 months post-treatment, and quality of life improved by 50–80% within a year. Tests were consistently shown to exhibit remarkable improvement in the severity of symptoms scores (SSSs) and scores for uterine fibroid symptom-health-related quality of life (UFS-QOL). Regarding fertility, pregnancy outcomes after HIFU varied from 16.7% to 38.8%, and the internal adenomyosis was associated with the best conception and natural pregnancy rates. Natural conception rates were several-fold higher for internal than for external adenomyosis (74.2% vs. 12.5%). Adjunctive hormonal therapy with LNG-IUS and GnRH agonists further enhanced symptom relief and long-term results. Safety reports indicated minimal re-intervention (2–8%) and recurrence (4–10%) rates. Side effects were largely mild (SIR Grade A/B) in nature, e.g., skin burns, hematuria (transitory), and sciatic nerve pain, while severe complications were rare. Notably, post-treatment AMH levels remained unchanged, suggesting preserved ovarian reserve. Rupture of the uterus was uncommon, but single case reports advise caution, particularly in the presence of additional risk factors or complicated pre-treatment protocols. The review highlights that the optimal outcome of HIFU is critically reliant on patient selection criteria—i.e., anterior lesion site, low vascularization, fewer number of hyperintense foci on MRI, and type III intramural adenomyosis—and reiterates that greater certainty regarding HIFU’s reproductive safety and efficacy must await larger, controlled trials [18].

#### 5.2.2. PMWA (Percutaneous Microwave Ablation)

PMWA is a thermotherapy that uses electromagnetic energy to kill adenomyotic tissue through the disturbance of water molecules, often by means of a transabdominal probe in general anesthesia. Compared to HIFU or radiofrequency ablation (RFA), PMWA is able to generate higher temperatures—often >100 °C—since it can propagate heat through desiccated or burnt tissue. The Asian Society of Endometriosis and Adenomyosis awarded it a Grade C recommendation [19]. Importantly, follow-up information about fertility or pregnancy outcomes following PMWA is not yet available.

#### 5.2.3. Radiofrequency Ablation (RFA)

RFA induces thermal injury to tumors by employing medium-frequency alternating electric currents, which generate local heat. The heat results in coagulative necrosis of the targeted tissue and also devastates the interstitial vasculature surrounding the tissue [20]. Similar to PMWA, RFA is acknowledged in the Asian Society of Endometriosis and Adenomyosis guidelines with a Grade C recommendation [19].

In a study by Nam, the use of ultrasound-guided transvaginal RFA was explored in the management of adenomyosis in relation to symptomatic improvement and fertility outcomes. The study had positive results, where 39 pregnancies were achieved among 29 women following RFA, recording a total pregnancy rate of 35.8%. Interestingly, 66.7% of the pregnancies resulted in live births, and the cesarean rate was 62.5%. There were no cases of uterine rupture, indicating the potential safety of this method for women desiring future fertility preservation. The relatively small number of patients and the lack of long-term obstetric outcome data, however, underscore the necessity of larger, prospective investigations to confirm these promising findings and establish the optimal selection criteria for patients [21].

#### 5.2.4. Uterine Artery Embolization (UAE)

UAE is a procedure that uses minimally invasive angiographic techniques to introduce embolic agents into the uterine arteries, with the goal of inducing ischemia in the adenomyotic tissue. The femoral artery is the most frequently used access point for the procedure, which is carried out under conscious sedation and a local anesthetic. The treatment causes hypoxia by obstructing arterial blood supply, leading to necrosis of the tissue within the lesion while preserving most surrounding uterine structures [22]. The UAE is mentioned in the guidelines from the Asian Society of Endometriosis and Adenomyosis, with a Level of Evidence 2 and a Grade of Recommendation B [19].

Serres-Cousine et al. documented 148 pregnancies and 109 live births (73.7% of pregnancies) among a substantial retrospective cohort of 398 women aged ≤ 43 years who underwent a fertility-sparing uterine artery embolization procedure. Of the live births, 74 were at term and 23 were preterm (average gestational age was 35.1 ± 2.78 weeks). The overall rate of pregnancy loss was 17.6%. Cesarean delivery was frequent (46.8% of live births) [23] (Table 2).

## 6. Comparative Analysis

A meta-analysis combined 13 studies that included nearly 800 women with adenomyosis who wanted to achieve fertility. Uterus-sparing conservative surgeries were followed by pregnancy rates of 40% after excisional treatment and 51% after non-excisional treatment, and live birth rates of 70% and 71%, respectively. The miscarriage rates were 21% and 22%, respectively. The difference between the two types of treatments was not statistically significant. Significantly, the incidences of reported uterine rupture were few or none across the studies, though the authors mention that most of the included studies were retrospective in design, employed non-standardized outcome measurements, and had a heterogeneous patient selection and surgical technique [10].

A retrospective analysis of 93 infertile women with adenomyosis compared HIFU (*n* = 50) with laparoscopic excision (LE) via adenomyomectomy (*n* = 43), showing major differences in reproductive outcomes. There was a higher clinical pregnancy rate in the HIFU group (52% compared to 30.2%, *p* = 0.034) and a higher spontaneous conception rate compared to the excision group (40% compared to 18.6%, *p* = 0.025). For the HIFU group, a 10% incidence of pregnancy-related complications was experienced with placenta accreta (4%), postpartum hemorrhage (4%), and a single incidence of preterm birth secondary to premature rupture of membranes (2%). There were fewer complications in the LE group, and these included two cases of fetal distress and one case of placenta accreta, all addressed via cesarean section without postpartum hemorrhage. Importantly, logistic regression analysis showed that adenomyosis location and type significantly influenced fertility outcomes after HIFU. Patients who had focal adenomyosis or posterior uterine wall lesions had significantly higher pregnancy rates than those with diffuse disease or anterior/lateral disease. These findings emphasize the need for precise lesion characterization to maximize patient selection and the individualization of conservative treatment regimens for optimized reproductive outcomes [24].

In a comparative trial of clinical efficacy and fertility in different conservative therapies of adenomyosis, ultrasound-guided percutaneous RFA with gonadotropin-releasing hormone analogs (GnRH-a) was superior. Among four groups—laparoscopic surgery alone, surgery plus GnRH-a, RFA alone, and RFA plus GnRH-a—the combined therapy (RFA + GnRH-a) group had maximum improvement in menstrual volume, dysmenorrhea, uterine volume, hormonal profiles, and CA125 levels. It also featured the highest clinical effectiveness rate (100%) and the highest pregnancy rate (74.3%), with higher spontaneous conceptions and fewer IVF cycles compared to other groups (*p* < 0.05) [25].

Moawad et al. conducted a narrative review of conservative surgical management for adenomyosis. Clinical pregnancy rates following uterus-sparing surgery ranged from 29.4% to 75%, and live birth rates ranged from 29.4% to 65.7%, differing by surgical technique and disease severity. Those with focal adenomyosis all had better outcomes compared to those with diffuse disease, with a study reporting a pregnancy rate of 75% in focal versus 43.2% in diffuse adenomyosis. Surgical interventions such as triple-flap or H-incision adenomyomectomy were commonly distinguished by higher spontaneous conception rates and fewer ART treatments. Notably, rupture of the uterus was rare, with single reports, but placenta accreta spectrum disorders were observed more frequently, particularly in patients who conceived after excisional surgery. The review further indicated that the inclusion of surgery with GnRH agonist treatment enhanced fertility and reduced the recurrence of symptoms [1].

## 7. Discussion

Underdiagnosed in reproductive-aged women in the past, adenomyosis is rapidly gaining recognition as a leading cause of subfertility and pregnancy morbidity [1,2].

There is mounting evidence to support the advantage of uterus-sparing surgical procedures like adenomyomectomy and non-excisional thermal ablation techniques as viable alternatives to improve reproductive outcomes. Excisional surgery, if conducted with meticulous lesion mapping and novel reconstructive techniques such as double-flap or triple-flap techniques, has superior outcomes, with conception rates >50% and live birth rates as high as 70% in well-selected groups [11,12]. Of interest are the theoretical advantages of surgical approaches over non-surgical treatments for focal adenomyosis patients versus diffuse disease, since it is presumably more technically possible to excise all lesion tissue and preserve more uterine integrity [1,4].

However, the benefits of excisional surgery must be weighed against potential obstetric risks. Although uterine rupture remains relatively rare, it has been reported in up to 6% of cases [13,14]. Furthermore, the risk of placenta accreta spectrum disorders also appears elevated following surgery, necessitating intensive preconception counseling and perinatal surveillance [13,14].

Unexpectedly, recent evidence suggests that non-excisional approaches such as HIFU and RFA yield comparable fertility outcomes with an improved safety profile. A large meta-analysis revealed no statistically significant variation in pregnancy, live birth, or miscarriage rates between excisional and non-excisional techniques [10]. HIFU, in particular, had excellent spontaneous conception rates and excellent symptom relief, but its live birth rate lagged behind that of surgery in some studies [16,17,18].

Adjuvant therapies—more specifically, postoperative hormonal therapy such as GnRH agonists—can enhance operative efficacy. For instance, combined treatment with RFA and GnRH agonists yielded the highest reported pregnancy rates (up to 74.3%) and best-harnessed hormonal and inflammatory markers in recent comparative studies [25]. Such findings favor a multimodal and individualized approach in treating adenomyosis infertility.

Despite growing optimism, several limitations continue across the current literature. The prevalence of retrospective investigations, small populations, and composite surgical procedures limits the generalizability of findings. In particular, most studies lack standardized diagnostic criteria (e.g., MUSA classification) and outcome reporting, and thus interstudy comparisons and evidence synthesis are difficult.

The absence of robust randomized controlled trials of surgery against medical or expectant management is a significant gap.

Furthermore, key predictors of reproductive outcomes—e.g., lesion location, patient age, CA125, and junctional zone thickness—are not additionally defined and codified. Their inclusion in standard preoperative assessments would significantly improve patient selection and outcome, particularly in the case of diffuse adenomyosis, where outcomes remain exceedingly variable.

## 8. Conclusions

This perspective review demonstrates that conservatively treated surgical management of adenomyosis can reliably improve fertility and pregnancy rates in appropriately selected patients. Excisional therapy, e.g., adenomyomectomy with modern reconstruction, is best for focal disease, with more than 50% pregnancy and more than 70% live birth rates. Non-surgical treatments like RFA and HIFU offer comparable fertility outcomes, especially in those with intracorporeal or mild disease, with essentially no risk of surgery. Of special note, adjunct hormonal therapy appears to contribute additional benefit, validating the logic of a multimodal approach. While these are positive outcomes, research evidence is liable to be diluted by study design heterogeneity, the absence of uniform diagnostic paradigms, and underreporting of diffuse adenomyosis cases. Moreover, the absence of recommendations from European professional societies makes it difficult to standardize the diagnostic and therapeutic approaches in clinical practice. By utilizing good-quality imaging, multidisciplinary opinions, and surgical expertise, uterine-sparing surgery can offer excellent reproductive potential, revolutionizing treatments for women afflicted with this condition’s clinical prognosis.

## Figures and Tables

**Table 1:** Practical aspects of each method.

Method	Technology Availability	Required Surgical/Operator Expertise
**Excisional Surgery (Adenomyomectomy)**	Widely available in advanced gynecologic centers	**High**: skills in advanced laparoscopic surgery, uterine reconstruction (triple-flap, overlapping, U-/H-suturing)
**UAE**	Available in hospitals with interventional radiology capabilities	**Moderate to High (radiology side)**: needs interventional radiologist with experience; gynecologic team coordination
**HIFU**	More limited; available in specialized centers	**Moderate**: operator must understand imaging guidance, patient selection (distance from skin, obstruction by bowel, etc.), safety (thermal effects)
**RFA**	Emerging; available in some centers; increasing interest	**Moderate**: needs training in use of ablation probes, imaging guidance, controlling ablation area

**Table 2:** Techniques and fertility outcomes, PE: preeclampsia, SGA: small-for-gestational-age.

Technique	Pregnancy Rate	Live Birth Rate	Cesarean Rate	Notable Findings	Reference
**Excisional Surgery (General)**	58.1%	46.9%	Not stated	Miscarriage: 8.9%; Preterm birth: 9.6%; No uterine ruptures	[11]
**Double-Flap Adenomyomectomy**	40.8%	72.6%	98.3%	Predictive factor: postoperative JZmax-A	[12]
**Excisional vs. No Treatment (Case–Control)**	Not specified	Not specified	Not specified	↓ PPROM, PE, SGA; ↑ placenta accreta (50%), 1 rupture (5.6%)	[13]
**Single-Surgeon Series (*n* = 22)**	Not specified	Not specified	100%	Preterm birth: 31.8%; Accreta: 2 cases; 1 rupture (4.5%)	[14]
**HIFU (Xiong et al.)**	37%	90% (Of Pregnancies)	50%	Mostly Spontaneous Conceptions; No Severe Delivery Complications	[16]
**HIFU (Meta-Analysis)**	53.4%	35.2%	33.2%	High Heterogeneity	[17]
**HIFU (Bahutair & Alhubaishi)**	16.7–38.8%	Not Specified	Not Specified	↑ Natural Conception in Internal vs. External (74.2% vs. 12.5%); Preserved AMH	[18]
**PMWA**	Not Available	Not Available	Not Available	No Fertility Data Available; Limited Clinical Endorsement	
**RFA**	35.8%	66.7% (Of Pregnancies)	62.5%	No Uterine Rupture; Promising but Needs More Data	[21]
**UAE**	37.2%	73.7% (Of Pregnancies)	46.8%	74 at term and 23 preterm	[23]

## Data Availability

Available from A.I. and N.M. upon reasonable request.
